# Monitoring of hemodialysis quality-of-care indicators: why is it important?

**DOI:** 10.1186/1471-2369-14-109

**Published:** 2013-05-24

**Authors:** Steven Grangé, Mélanie Hanoy, Frank Le Roy, Dominique Guerrot, Michel Godin

**Affiliations:** 1 Nephrology department, Rouen University Hospital, 1 Avenue de Germont 76031 Rouen Cedex, Rouen, France; 2 INSERM Unit 1096, Rouen University Medical School, Rouen, France

**Keywords:** End-stage renal disease, Guidelines, Hemodialysis, Morbidity, Quality-of-care indicators

## Abstract

**Background:**

Meeting specific guideline targets is associated with improved survival rates and reduced hospitalizations in the dialysis population. This prospective work evaluated the adequacy of hemodialysis quality indicators in an in-center hemodialysis population with severe comorbidities, and assessed whether clinical practice could impact intermediate outcomes.

**Methods:**

All the chronic hemodialysis patients treated in Rouen University Hospital hemodialysis Unit between January 2009 and April 2010 were included in this observational study. Every quarter, mean levels and prevalence of conformity were collected for the following indicators: anemia, dialysis dose, serum calcium and phosphorus, PTH, 25OH-vitamin D, albumin, serum bicarbonate, LDL-cholesterol, serum β2-microglobulin, systolic and diastolic blood pressure, intradialytic hypotension and vascular access. Conformity of quality-of-care indicators was determined according to targets defined by international guidelines, whenever available.

**Results:**

Altogether, 124 patients were included in the study. Thirty-three patients were evaluated during the entire follow-up period. An improvement in the percentage of conformity was observed for hemoglobin, dialysis dose, phosphates, PTH, serum bicarbonate and β2-microglobulin in the global population. Failure to improve conformity rates for several indicators, including serum albumin, was found, possibly depending on patients’ comorbidities rather than on quality of care.

**Conclusion:**

Overall, this study shows that following quality-of-care indicators can improve clinical practice by identifying center-specific weaknesses, prompting the establishment of corrective measures. Finally, we suggest that the definition and targets of some indicators, especially hypertension and LDL-cholesterol, be reviewed, since evidence of their association with mortality is not demonstrated.

## Background

Hemodialysis patients present persistent high morbidity and mortality rates, in spite of the promising technical advances developed over the last 15 years. In Europe, survival rates of patients who begun hemodialysis between 2002 and 2006 were 78.7% and 65.8% after 1 year and 2 years, respectively [1]. However, survival improved by 10%, between the patients who started hemodialysis in 1997–2001 and those who started in 2002–2006. This improvement, despite increases in the age and prevalence of diabetes, mainly reflects the relative importance of the quality of care.

Two large observational studies in prevalent and incident patients have shown that the increasing number of unfulfilled therapeutic targets was associated with higher mortality and hospitalization rates [2,3]. The main quality-of-care indicators are well defined [4-6]. Numerous studies have documented that an increased risk for death and hospitalization was associated with lower levels of dialysis adequacy, increased anemia, lower serum albumin values, and the use of a vascular access other than an arteriovenous fistula (AVF) for hemodialysis. Consequently, clinical practice guidelines such as the Kidney Disease Outcomes Quality Initiative (KDOQI) or the European Best Practice Guidelines (EBPG) were developed in order to improve the quality of care and outcomes of hemodialysis patients [7].

The aim of this prospective study was to analyze the monitoring of established quality-of-care indicators in an in-center hemodialysis population, and to identify the indicators that were not achieved. When it was necessary, a centre-specific intervention was decided, to improve our results concerning these indicators. We critically analyze these results and the impact of monitoring quality-of-care indicators in our clinical practice. Based on these practical issues, we suggest alternative quality-of-care indicators that can easily be monitored, and may be relevant because of their association with morbidity and mortality in large observational studies.

## Methods

This monocentric prospective study included every prevalent and incident patient admitted in Rouen University Hospital Hemodialysis Unit, from January 2009 through April 2010. Patients with acute renal failure were excluded.

All patients received dialysis with a Fresenius 5008 dialysis machine (Fresenius Medical Care, Bad Homburg, Germany) and biocompatible high-flux membranes (Kuf > 40 ml/h/mmHg) with surface area above 1.8 m^2^. Prevalent patients had an AVF every time it was possible. For incident patients, tunnelized catheters were placed in the days following the initiation of hemodialysis and were converted to AVF as soon as possible. The traditional regimen consisted of 4 hours dialysis sessions, three times a week. Patients were treated with conventional hemodialysis or hemodiafiltration. Blood flow rate and dialysate flow rate were 400 ml/min and 800 ml/min, respectively. The dialysis dose (Kt) was available for each seance via the on-line clearance monitoring module (OCM), measuring ionic dialysance. The volume of distribution of urea was estimated using bio-impedancemetry (BCM; body composition monitor, Fresenius Medical care, Bad Homburg, Germany) [8]. For the patients on hemodiafiltration, the infusion volume was also collected. Isothermic dialysis, by means of a blood temperature monitor (Module BTM, available on Fresenius 5008 machines), and isonatremic dialysis were systematically performed [9,10]. A dialysate calcium of 1.5 mmol/l was used for the majority of patients, while 1.75 mmol/l was never used. The bicarbonate content of the dialysate was individualized and adjusted to achieve a pre-dialysis bicarbonate level between 20 and 24 mmol/l.

When malnutrition was diagnosed, patients received nutritional counselling from a qualified dietician. Nutrition supplements were prescribed if nutritional counselling did not achieve an increase in nutrient intake to a level covering minimum recommendations. Intradialytic parenteral nutrition was prescribed in the patients who experienced dietary support and/or oral supplement failure, in particular in hospitalized patients with acute inflammatory state or inflammatory bowel disease (Smofkabiven 1100 kcal, Fresenius Kabi, France, the dose was increased at 1600 kcal per dialysis session in case of good tolerance). All patients received calcifediol once a week, the dose depending on the severity of 25OH-vitamin D deficiency. Statins were systematically prescribed.

For patients with high blood pressure (BP > 140/90 mmHg) despite antihypertensive multitherapy, after reduction of the dry weight when necessary, ambulatory blood pressure monitoring was performed to confirm hypertension before prescribing an additional antihypertensive agent.

Changes in the prescription of erythropoiesis stimulating agents (ESA), phosphate binders and bicarbonate dialysate concentration were carried out every month in parallel. Before adjusting ESA, intravenous iron supplementation was performed, if needed, to reach a ferritinemia between 200 and 500 μg/l and transferrin saturation > 20%.

The following indicators were collected monthly: Hemoglobin, ESA dose, serum phosphorus, calcium, albumin and bicarbonate concentrations. Unless specified, the following indicators were recorded every quarter:

Serum parathyroid hormone, serum 25OHvitD (every 6 months), serum β2-microglobulin.

Total cholesterol and LDL-cholesterol.

Technique of renal replacement therapy: hemodialysis, post-dilution on-line hemodiafiltration, pre-dilution on-line hemodiafiltration, daily post-dilution on-line hemodiafiltration, hemofiltration.

Pre-dialysis systolic and diastolic BP of the 10 last dialysis sessions. The mean of the 10 values was used to determine whether or not the target was achieved.

Dialysis dose: On-line urea clearance estimation makes it possible to calculate the dialysis dose Kt and thus allows for the estimation of the « single-pool » Kt/V for each session [8]. Kt/V_BCM_ of the 10 last dialysis sessions were collected. The mean of the 10 values was used to determine whether or not the target was achieved (Kt/V_BCM_ > 1.4).

Percentage of dialysis sessions with symptomatic intradialytic hypotension (IDH), defined by a decline in BP associated with specific symptoms, with the need to stop ultrafiltration and/or saline infusion, taking into account the last 10 sessions.

Prevalence of conformity was defined by the percentage of patients who attained targets for each indicator. Every quarter, the mean value and the prevalence of conformity for each quality-of-care indicator were calculated in the global population and in the patients who remained in the center between January 2009 and April 2010.

This study did not require ethical approval according to French research legislation.

Targets, shown in Table 1, were defined by international guidelines including KDOQI and EBPG guidelines, and by evidence available in the literature in the absence of existing guidelines [5,11-13]. When conformity rates were below those found in guidelines or literature, a decision was made to initiate corrective measures. For the main indicators, quarterly meetings were organized, where individual corrective measures were decided for the patients who did not attain the target.

**Table 1:** Quality-of-care indicators, targets and references

**Field**	**Clinical indicator**	**Frequency**	**Clinical performance measures**	**References**
**Anemia**	Hemoglobin (Hb, g/dl)	M	% of patients with 10 < Hb < 13	*
**Dialysis dose**	Kt/V_BCM (single-pool)_	S	% of patients with Kt/V_BCM_ > 1.4	EPBG 2002
	Kt (liters)	S	% of patients with Kt > 40l (women), with Kt > 45l (men)	Lowrie et al. [14-16]
**Bone metabolism**	Phosphorus (mmol/l)	M	% of patients with 1.13 < P < 1.78	KDOQI Bone metabolism 2003
	Calcium (mmol/l)	M	% of patients with 2.10 < Ca < 2.38	KDOQI Bone metabolism 2003
	PTH (pg/ml)	Q	% of patients with 150 < PTH < 300	KDOQI Bone metabolism 2003
	25(OH)vitD (nmol/l)	Q	% of patients with 72 < vitD < 200	*
**Nutrition**	Albumin (g/l)	M	% of patients with albumin > 35	*
	Serum Bicarbonate (mmol/l)	M	% of patients with 20 < HCO3^-^ < 24	*
	LDL-cholesterol (mmol/l)	Q	% of patients with LDL < 2.6	*
**Vascular access**	Arteriovenous fistula		% of patients with catheters < 7%	Dopps [4]
**Middle molecule removal**	Infusion Volume (Liters)	S	Infusion volume > 15 L	*
	β2-microglobulin (mg/l)	Q	% of patients with β2m < 27.5	Cheung et al. [17]
**Hemodynamics**	Blood pressure	S	% of patients with pre-dialysis BP < 140/90 mmHg	KDOQI 2005 [5]
	Hemodynamics instability		% of dialysis sessions with symptomatic IDH	

: Targets different from available clinical practice guidelines.

Frequency : S = Values collected each dialysis session ; M = monthly ; Q = quarterly.

Statistical analysis: Comparison between initial (January 2009) and follow-up (April 2010) conformity rates was made using chi-square analysis. P < 0.05 was considered to indicate significance.

## Results

Overall, 124 patients were included in the study. 33 patients were evaluated during the entire follow-up period. Demographic data are listed in Table 2.

**Table 2:** Characteristics of the study population (n = 124) Values are n (%), unless otherwise specified

**Age (mean ± SD)**	**69.1 ± 14**
Men	72 (58%)
Women	52 (42%)
Time on hemodialysis (mean ± SD)	32.6 ± 42
**Comorbidities**	
Diabetes Mellitus	59 (47.6%)
Hypertension	99 (79.8%)
Dyslipidemia	71 (57.3%)
Ischemic Cardiomyopathy	50 (40.3%)
Lower Limb arteriopathy	31 (25%)
Charlson Score (mean ± SD)	8 ± 2.6
**Renal Disease**	
Diabetes Nephropathy	35 (28.2%)
Vascular-Hypertensive	29 (23.4%)
Polycystic-kidney disease	5 (4%)
Glomerular	12 (9.7%)
Others	43 (34.7%)

### Quality indicators

**Figure 1:**
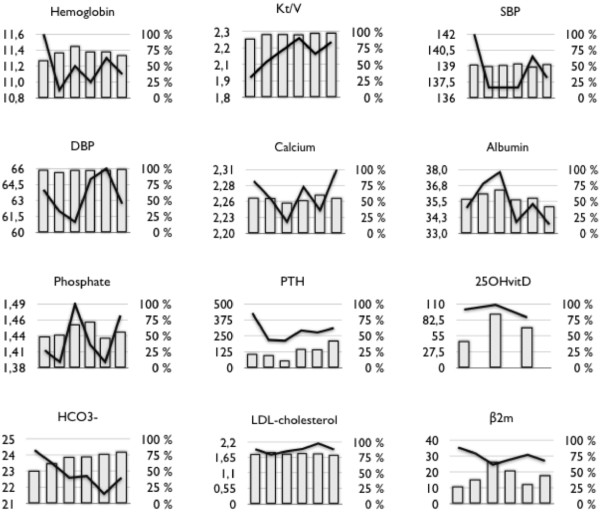
**Quality indicators: Evolution of mean levels (lines) and conformity rates (histograms) during the 6 quarters.** Hemoglobin (g/dl) ; SBP and DBP : Systolic and Diastolic Blood Pressure (mmHg) ; Serum Calcium (mmol/l) ; Serum Albumin (g/l) ; Serum Phosphate (mmol/l) ; PTH (pg/ml) ; 25OHvitaminD (nmol/l) ; Serum Bicarbonate (mmol/l) ; LDL-cholesterol (mmol/l) ; β2-microglobulin (mg/l).

Table 3 shows the results corresponding to the time-points of January 2009 (baseline) and April 2010 (follow-up, after corrective measures) in the total population and in the 33 patients who were hemodialyzed in our center during the entire study period. The trend for each indicator was the same whether or not the incident patients were being taken into account in the analysis. For each indicator, evolution of mean levels (lines) and conformity rates (histograms) during the 6 quarters are shown in Figure 1.

**Table 3:** Quality indicators: mean levels and conformity rates

	**All patients January 2009**	**All patients April 2010**	P value*	**33 patients January 2009**	**33 patients April 2010**	**P Value**
**N patients**	65	69		33	33	
**Sex ratio**	1.09	1.33		0.89	0.89	
**Dialysis Vintage (months)**	41.3	40		54.7	68.5	
**Dialysis technique**	78,5%	37.1%		67.6%	8.8%	
**Hemodialysis**	0%	1.4%		0%	0%	
**Hemofiltration**	4,6%	4.3%		5.9%	5.9%	
	13,8%	52.9%		20.6%	85.3%	
**Daily HDF**						
**Post-dilution HDF**	3,1%	4.3%		5.9%	0%	
**Pre-dilution HDF**						
**Hemoglobin**	11.3/55.4%	10.9/65.2%	NS	11/50%	11,1/67,6	0.03
**EPO doses UI/Kg/week**	111.3	133.8		106	126.4	
**Albumin (g/dl)**	35/53.1%	34/44.9%	NS	34,7/52,9%	33,6/38,2%	NS
**Calcium (mmol/l)**	2.28/58.4%	2.30/60.8%	NS	2,27/47%	2,30/47%	NS
**Phosphates (mmol/l)**	1.43/44.6%	1.48/60.3%	0.008	1.36/44%	1.45/55.9%	NS
**PTH (pg/ml)**	442/22%	315/43.7%	0.0005	389/14.7%	271/35.3%	0.01
**25OHvitD (nmol/l)**	94.5/43%			94.9/67.6%		
**HCO**^**3- **^**(mmol/l)**	24/50.8%	22.7/81.1%	<0.0001	24.3/50%	22.7/82.4%	<0.0001
**LDL-cholesterol (mmol/l)**	1.99/79.4%	1.95/80%	NS	2.1/76.5	1.95/70.6%	NS
**B2microglobulin (mg/l)**	34.78/28.2%	26.3/48.5%	0.001	32.6/29.4%	28.5/38.2%	NS
Pre-dialysis SBP (mmHg)	142/52.3%	138.4/51.4%	NS	138.9/58.8%	137.3/58.8	NS
Pre-dialysis DBP (mmHg)	64/98.5%	62.3/100%	NS	62.3/100%	63.3/100%	NS
Kt (L)	55.6/95.1%	60.1/97%	NS	57.2/88.2%	63.6/97%	0.002
**Kt/V**	1.94/90%	2.19/98.4%	<0.0001	2.07/85.3%	2.37/94%	0.03
**Vascular access (catheters < 7%)**	78.5% AVF	74.3% AVF	NS	85.3% AVF	88.2% AVF	NS
	21.5% catheter	25.7% catheter		14.7% catheter	11.8% catheter	
% of dialysis sessions with IDH	11.8%	12.2%	NS	9.1%	10.6%	NS

*: Comparison of the conformity rates. A p value < 0.05 was considered statistically significant.

We compared serum phosphorus between the patients treated by haemodialysis (n = 17, mean phosphorus: 1.61 mmol/l) and those treated by post-dilution haemodiafiltration (n = 29, mean phosphorus: 1.41 mmol/l) between January and April 2010. We observed a trend for lower serum phosphorus levels in the « hemodiafiltration » group compared to the « hemodialysis » group (p = 0.09).

## Discussion

CKD is associated with increased mortality, mainly attributable to cardiovascular events. In ESRD patients, optimization of dialysis quality and cardiovascular risk factors is consequently a major issue, and monitoring of specific indicators is therefore mandatory. A relevant quality-of-care indicator should have two main characteristics: it should be associated with a lower risk of death, and attainment of the target should be possible thanks to medical practice changes. In this study over a 12-month period, an improvement in the percentage of conformity to predefined targets was observed for hemoglobin, dialysis dose, phosphates, PTH, serum bicarbonate and β2-microglobulin.

### Evidence-based quality-of-care indicators

#### Anemia management

The percentage of patients who achieved a Hb level within the 10–13 g/dl target range increased from 60% to 80%, which was in accordance with the results obtained from the national data system « REIN 2008 ». This improvement may be due to the increase of erythropoietin doses and to the optimization of the iron status. In terms of mortality, meta-analyses did not show any statistically significant difference between higher and lower Hb level in hemodialysis patients [18]. This target allows for relative flexibility in medical decision making and takes into account variability between patients’ comorbidities, prognosis, functional status, and responsiveness to ESA therapy. In in-center hemodialysis patients, it is more difficult to achieve hemoglobin targets because of comorbidities, including numerous diseases with inflammatory state, responsible for ESA resistance. Accordingly, we found no correlation between erythropoetin doses and the percentage of conformity for anemia.

#### Vascular access management

Our percentage of patients hemodialysed with catheters did not decrease. Indeed, the majority of incident patients did not start dialysis with AVF. Our conformity rate for vascular access was higher than 80% in 2009 when we excluded the incident patients who had initiated hemodialysis for less than three months, whereas it was lower than 80% when these patients were included in the analysis. Another reason is that there is a lack of suitable vessels to create AVFs because of an aging and diabetic population. Data from « REIN 2008 », that include both in-patients and out-patients, showed that 16.5% of 22852 patients were hemodialysed via catheters in France in 2008. In a cohort study of 78420 maintenance hemodialysis patients comprising approximately 26% of the US hemodialysis population, Lacson Jr *et al.* found a 39% increased risk of death with catheters compared with fistulas, making the vascular access the second most important actionable variable associated with mortality after albumin [17]. Thus, the first measure to improve our percentage of patients dialysed on AVF would be to avoid the late referral of the patient to the nephrologist, which has been associated with increased catheter use. The second would be to shorten the delay before the creation of the AVF after initiation of dialysis.

#### Dialysis dose management

The prevalence of conformity for dialysis dose increased regularly, up to 98.4% in April 2010. The few patients who did not reach the Kt/V_BCM_ target had either a high urea distribution volume, or a dysfunction of their vascular access (catheter dysfunction or immature fistula). Results from « REIN 2008 » showed that 78.4% of 13451 patients had a Kt/V > 1.2. We used high blood and dialysate flow rates, membranes with a large surface area, and performed high-flux dialysis according to the results of the HEMO Study [19]. The prevalence of patients using hemodiafiltration increased during follow-up, which is a potential explanation for these results. Indeed, small solute dialysis dose delivered by hemodiafiltration is higher than that delivered by hemodialysis, because of increasing convective clearance. Canaud *et al.* showed that high-efficiency hemodiafiltration (infusion volume > 15 liters) had a positive impact on survival compared to patients treated by high-flux hemodialysis [20]. Articles suggest that dosing of dialysis should be based on the volume of blood cleared (Kt), rather than on Kt/V, which can lead to under-dialysis in women and small men by underestimating the hemodialysis dose [14,15,21]. Kt can easily be monitored during each treatment with the OCM device.

### Non evidence-based quality-of-care indicators

#### Hypertension management

Hypertension is a major cardiovacular risk factor in ESRD patients. In our study, the prevalence of conformity for systolic blood pressure was not improved. However, hypertension guidelines in hemodialysis patients are not currently based upon evidence. KDOQI guidelines concerning blood pressure target ranges were extrapolated from the general population [5]: Predialysis and postdialysis BP goals should be <140/90 mm Hg and <130/80 mmHg respectively, provided that there is no substantial orthostatic hypotension and that these levels are not associated with substantial and symptomatic intradialytic hypotension. Tentori *et al.*, in a retrospective analysis in 13792 incident hemodialysis patients, showed that following the guideline for predialysis blood pressure (BP) measurements was associated with increased mortality [5]. Zager *et al.* found a « U » curve relationship between post-dialysis SBP and cardiovascular mortality in more than 5400 hemodialysis patients. SBP < 110 mmHg and > 180 mmHg, and DBP > 90 mmHg were associated with poor outcomes [16]. Indeed, relative hypotension is a potent marker of mortality in ESRD patients, probably reflective of cardiac failure. Thus, it is unclear which BP target should be used as a quality-of-care indicator for hemodialysis patients. In the future, targets related to ambulatory BP measurements (ABPMs) or home BP measurements may be used. ABPMs were found to be superior to dialysis unit recordings in predicting outcomes [22]. In a prospective cohort study conducted in 150 chronic hemodialysis patients, self-measured and ambulatory systolic BP between 125 and 145 mmHg, and between 115 and 125 mmHg, respectively, were associated with a decreased risk of death [23].

#### Nutrition management

No improvement was observed in the mean serum albumin level and the percentage of conformity, which is around 50%. Our results were different from those reported in « REIN 2008 », where 64.9% of 24436 patients had an albumin > 35 g/l, presumably because of increased comorbidities in our in-center patients.

Albumin level showed the strongest association with mortality compared with other predictor variables in several large observational studies [24]. However, when adjusted to other major comorbidities, hypoalbuminemia was not significantly associated with mortality [25]. Since comorbid medical conditions may decrease albumin synthesis in the liver, hypoalbuminemia is a non-specific marker of denutrition and a difficult-to-modify patient factor, better associated with patient comorbidities than with poor quality of care [26]. In a randomized controlled study involving 180 patients with albuminemia below 37 g/l, a nutrition intervention tailored to patient-specific barriers resulted in modest improvements in albumin levels, regardless of levels on inflammatory markers [27]. In our study, albumin was measured by immunonephelometry, which is currently considered as the gold-standard method. The threshold used by the KDOQI (40 g/l) to detect hypoalbuminemia was not chosen because the bromocresol-green method used for KDOQI guidelines overestimates albuminemia [28].

The conformity rate for serum bicarbonate increased from 50.8 to 81.1% during the study period, due to the individualized prescription of bicarbonate dialysate concentration. The EBPG guidelines recommend that the mid-week predialysis serum bicarbonate levels should be maintained at 20–22 mmol/l [13]. A target range between 20 and 24 mmol/l was used in this study. A U-shape relationship between serum bicarbonate and mortality or hospitalization has been demonstrated in hemodialysis patients in the DOPPS study [29]. After adjustment for numerous comorbidities and for nutritional markers, the lowest risk for mortality was observed with serum bicarbonate between 20.1 and 21 mmol/l and the lowest risk for hospitalization was observed for serum bicarbonate levels between 21.1 and 22 mmol/l. In this study, there was an inverse correlation between pre-dialysis serum bicarbonate and albuminemia, suggesting that the lower bicarbonate concentrations resulted from greater acid load caused by protein intake. Moderate predialysis acidosis may be associated with better nutritional status, which is strongly associated with increased survival in hemodialysis patients. Patients with persistent and severe metabolic acidosis may be treated by increasing the bicarbonate dialysate concentration or with oral sodium bicarbonate. Patients with serum bicarbonate levels higher than the upper target range can be treated by lowering the bicarbonate dialysate concentration, bearing in mind that intradialytic alkalosis is poorly tolerated with more hypotensive episodes during the dialysis sessions [30].

#### Dyslipidemia management

Using dyslipidemia to define a quality-of-care indicator is unlikely to be clinically relevant, for several reasons. First, there is an inverse association of total cholesterol with mortality in dialysis patients [31], probably due to the cholesterol-lowering effect of systemic inflammation and malnutrition. Secondly, the only two large trials that studied the effects of statins in haemodialysis patients did not show any beneficial effect on cardiovascular death [32,33]. In a *post-hoc* analysis of the 4D trial (*Die Deutsche Diabetes Dialyse* Study), high levels of LDL-cholesterol showed a tendency to increase the risk of cardiac endpoints and all-cause mortality. In patients with a baseline LDL-cholesterol greater than 1.45 g/l atorvastatin significantly decreased adverse fatal and non-fatal cardiac events and all-cause mortality, compared to placebo. As low serum cholesterol levels are associated with increased mortality, this indicator was not chosen as a « quality-of-care » indicator. Maybe cholesterol should be used to identify patients who need statins, *ie* patients with high LDL-cholesterol (> 1.45 g/l). Of course, statins should be continued in patients who already had this treatment before initiation of dialysis for a cardiac event [32].

#### Bone metabolism

The conformity rate for serum calcium targets was stable around 60% during the study period despite the use of calcimimetics or low dialysate calcium concentration; it was above that observed in the national data system for bone metabolism (52%, Observatoire Photo-graphe ; n = 11172 patients) [34]. Recent KDIGO guidelines introduced new targets [12]. Despite a lack of evidence from randomized controlled trials demonstrating that attaining serum calcium targets impacts clinical outcome, large observational studies showed that the inflection point at which calcium becomes associated with an increased relative risk of all-cause mortality varies among studies, from 2.38 to 2.85 mmol/l [35-37]. In an observational study in incident hemodialysis patients, hypocalcemia < 2.20 mmol/l was independently associated with mortality (RR 2.10) [38]. Chronic hypocalcemia was significantly associated with both *de novo* and recurrent ischemic heart disease, and *de novo* and recurrent cardiac failure. Thus, the KDIGO lower and upper thresholds for calcium could be adapted, but one may argue that with this new target range, the mean serum calcium level will increase, and that high levels of Ca-P product may favor vascular calcification [39].

In France, the conformity rate for serum phosphorus and the mean serum phosphate level were 52% and 1.56 mmol/l in June 2009, respectively (Observatoire Photo-graphe data). The mean serum phosphate level was lower in our study, probably because of the increased prevalence of malnutrition. The percentage of conformity for serum phosphorus improved during the follow-up, up to 72.3% in October 2009. The wider use of hemodiafiltration may partially explain this result, since we observed a trend towards lower serum phosphorus in hemodiafiltration patients. Two large prospective observational studies showed that this dialysis modality could improve phosphate control [40,41]. In the study of Lars Penne *et al.*, the proportion of hemodiafiltration (HDF) patients with pre-dialysis phosphate concentrations < 1.78 mmol/l increased from 64% to 74% during the 6-month study period and was stable in hemodialysis patients. Nevertheless, two RCTs did not show any benefit of HDF on phosphate control, maybe because of the low baseline phosphate levels (1.58 mmol/l et 1.62 mmol/l) [42,43]. There is no evidence from RCTs that lowering serum phosphorus to a specific target range reduces mortality in hemodialysis patients. The target range for phosphorus was modified with the publication of the KDIGO clinical practice guidelines. The current target is between 0.9 and 1.5 mmol/l [12]. Our conformity rate will decrease with this new target range. Thus, more aggressive strategies will have to be adopted to lower serum phosphorus, such as adding a convective component to clearance with hemodiafiltration, lengthening dialysis session time or increasing dialysis frequency.

#### Vitamin D management

Because of the limited sunlight exposure in Normandy, our patients are systematically supplemented with 25OHvitD. Nevertheless, our conformity rate for vitamin D was not satisfactory, with important variations considering the mean vitamin D levels. Increasing evidence sugsests that Vitamin D deficiency is an independant risk factor for cardiovascular events and all-cause mortality in hemodialysis patients [44,45]. In the 2009 KDIGO guidelines, repeated measurements of 25OHvitD and therapeutic supplementation in case of deficiency are recommended [12]. A randomized controlled trial should be performed to clarify whether vitamin D supplementation can decrease adverse outcomes. Thus, vitamin D may become an important quality-of-care indicator in the near future.

#### Parathormone

In France, the conformity rate for PTH and the mean serum PTH level were 33% and 317 pg/ml in June 2009 respectively (Observatoire Photo-graphe data), whereas prevalence of conformity for PTH increased from 22% to 42.8% in our study. According to recent KDIGO guidelines PTH levels should be maintained in the range of approximately two to nine times the upper normal limit for the assay (45 to 490 pg/ml) [12]. Observational data demonstrated that the K/DOQI treatment goals were not easily achieved or maintained with traditional therapeutic options (phosphate binders, vitamin D analogs) for secondary hyperparathyroidism (SHPT). Moe *et al.* performed a secondary analysis of three large RCTs, demonstrating that cinacalcet effectively reduces PTH, calcium, and phosphorus to the K/DOQI target ranges in hemodialysis patients with SHPT [46]. In 2010, Block *et al.* showed in a prospectively designed observational study a significant survival benefit associated with prescribing cinacalcet for hemodialysis patients with evidence of SPHT and receiving i.v. vitamin D [47]. In contrast, the recently published EVOLVE study found no benefit to cinacalcet in hemodialysis patients and raised significant safety issues [48].

#### Middle molecule removal management

An important improvement concerning the conformity rate for β2M was noticed, which increased from 28.2% to 48.5%. β2-microglobulin (β2M) is a marker for middle molecules in uremia and a potential target for adequacy in hemodialysis therapy. In 1704 patients from the HEMO study, pre-dialysis serum β2M levels > 27.5 mg/l were associated with all-cause-mortality [49]. Since we increased the use of hemodiafiltration with ultrapure dialysate, which is the most efficient therapy to reduce serum β2M levels, we expected a better conformity rate for β2M. Indeed, convective treatments are an established therapy to enhance uremic toxin removal over a wide molecular-weight spectrum. Maduell *et al.* found mean β2-microglobulin reduction rates were 75.4% for on-line post-dilution hemodiafiltration versus 60.1% for high-flux hemodialysis [50]. They also showed that short daily on-line hemodiafiltration was associated with lower pre-dialysis serum β2-microglobulin levels [51]. In addition, dialyzers with better β2M clearance may be a therapeutic option, although there may be an associated risk of greater albumin loss.

#### Intradialytic hypotension management

The percentage of dialysis sessions with intradialytic hypotension (IDH) remained low during the study period, around 12%. Mortality in patients with frequent IDH is significantly higher than in those without such events, but after adjustment for covariates, this association loses significance [52]. Thus, IDH may represent a marker of comorbid conditions. Our in-center dialysis study failed to demonstrate a substantial improvement for this indicator, despite many measures systematically followed to improve hemodynamic instability, perhaps because it was already very low. The dry weight was assessed by clinical examination with the help of bioimpedance measurements, using the BCM. We checked in those patients with frequent IDH that sodium restriction and timing of antihypertensive agents were well respected. More than 90% of the patients during the study period were treated with isothermic dialysis, that was shown to improve hemodynamic instability, as well as dialysis at cooler dialysate temperatures [9,53]. The percentage of patients on on-line post-dilution hemodiafiltration increased during the study. This convective technique was associated with a significant reduction of hypotensive episodes, predominantly related to decreased body temperature [54]. In the EBPG guidelines on hemodynamic instability, convective techniques are a possible alternative to cool dialysis. If these treatment options have failed, other available therapies are suggested: midodrine (level 1 evidence), blood volume controlled ultrafiltration, use of a dialysate calcium of 1.5 mmol/l, prolongation of dialysis time or increase in dialysis frequency (level 2 evidences), L-carnitine supplementation, and/or bicarbonate dialysis (level 3 evidences) [55].

The importance of quality-of-care indicators is well established. Some indicators, as defined by international guidelines, rely on a solid scientific basis with clear associations with outcomes (hemoglobin, albumin, dialysis dose, vascular access). Nevertheless, the clinical advantage of meeting multiple treatment targets simultaneously remains to be established. In addition cost/benefit evaluations of each indicator, and especially of strategies based on multiple quality-of-care indicators should be performed. In hemodialysis patients, the association between several indicators, such as phosphate, BP or BMI, and mortality is characterized by a J-shaped curve. In this context, which quality-of-care indicator should be prioritized may be a matter of debate. In addition, regarding several routinely measured parameters, such as 25OHvitaminD or β2-microglobulin, evidence for specific targets and guidelines are lacking, suggesting that locally determined targets may be useful before RCTs and evidence-based guidelines are available.

## Conclusions

In conclusion, this study shows that following quality-of-care indicators can improve clinical practice by highlighting center-specific weaknesses, prompting the establishment of corrective measures. Our work points out the difficulty of using standardized targets for quality-of-care indicators. We suggest that indicators based on scientific evidence should be prioritized, and that the definition and targets of some indicators, especially hypertension and LDL-cholesterol, be reviewed, since evidence of their association with mortality is not clearly demonstrated.

## Abbreviations

ABPM: Ambulatory blood pressure measurements; AVF: Arteriovenous fistula; β2M: β2-microglobulin; BCM: Body composition monitor; BP: Blood pressure; BTM: Blood temperature monitor; CKD: Chronic kidney disease; EBPG: European best practice guidelines; ESA: Erythropoiesis stimulating agents; ESRD: End-stage renal disease; HDF: Hemodiafiltration; KDOQI: Kidney disease outcomes quality initiative; IDH: Intradyalytic hypotension; OCM: On-line clearance monitoring module; SPHT: Secondary hyperparathyroidism

## Competing interests

No financial or non-financial competing interest is declared.

## Authors’ contributions

SG performed a clinical interpretation of the data and wrote the manuscript. MH and FLR collected the major part of the data, performed the statistical analysis, contributed to the discussion and reviewed the manuscript. DG and MG reviewed the manuscript. All authors read and approved the final manuscript.

## Pre-publication history

The pre-publication history for this paper can be accessed here:

http://www.biomedcentral.com/1471-2369/14/109/prepub
